# Mitochondrial Labeling with Mulberrin-Cy3: A New Fluorescent Probe for Live Cell Visualization

**DOI:** 10.3390/bios14090428

**Published:** 2024-09-05

**Authors:** Gangxiang Yuan, Yiwei Luo, Peng Qian, Ningjia He

**Affiliations:** State Key Laboratory of Resource Insects, Southwest University, Chongqing 400715, China; yuangx99@email.swu.edu.cn (G.Y.); luoyiwei12@swu.edu.cn (Y.L.); qp125678@email.swu.edu.cn (P.Q.)

**Keywords:** mitochondria, mulberrin-Cy3, fluorescent labeling, probe

## Abstract

Mitochondria, crucial intracellular organelles, are central to energy metabolism, signal transduction, apoptosis, calcium homeostasis, and a myriad of other biological processes, making them a focal point in diverse research fields. The capacity to fluorescently label and visually track mitochondria is crucial for understanding their biological roles. We present mulberrin-Cy3, a novel small molecule fluorescent probe that selectively labels mitochondria in animal cells, including cancer cells, with relative ease. This protocol details the synthesis of mulberrin-Cy3 and its use for visualizing mitochondria in living cells. The synthesis is straightforward and time-efficient, and the labeling method is more accessible than traditional approaches, providing a cost-effective option for mitochondrial visualization at room temperature. The labeling is rapid, with effective labeling achieved within 5 min of incubation. The fluorescent signal is stable and brighter, offering a significant advantage over existing methods. Mulberrin-Cy3 represents a promising mitochondrial labeling compound, providing researchers with a novel experimental tool to explore the complex biological functions of mitochondria. This innovation has the potential to significantly advance our comprehension of mitochondrial dynamics and their role in cellular health and disease.

## 1. Introduction

Mitochondria are important organelles within cells, involved in numerous cellular processes and possessing multiple functions [1]. The most recognized role of mitochondria is their contribution to ATP production, which is crucial for sustaining various cellular activities [2]. Mitochondria are also crucial for the TCA cycle and are closely linked to fatty acid β-oxidation [3]. Research indicates that mitochondria regulate various metabolic processes [4,5], including amino acid metabolism [6], the urea cycle [7], and steroid synthesis [8]. Additionally, mitochondria maintain intracellular calcium ion homeostasis by storing and releasing calcium ions [9,10]. They are also closely associated with apoptosis [11] and are the main source of reactive oxygen species (ROS) production [12]. Mitochondrial biogenesis and dynamics are essential for preserving mitochondrial function, distribution, and quality control [13]. Beyond these core functions, mitochondria participate in the biosynthesis of heme, iron-sulfur clusters [14], certain amino acids [6], and nucleotides [15], as well as in various cellular signaling pathways related to stress responses [16], inflammation [17], and hypoxia regulation [18].

To elucidate the function and mechanism of action of mitochondria in various roles, labeling mitochondria within cells is essential to studying their structure, function, and dynamics. Over the years, researchers have developed various methods to label cellular mitochondria. These include: (1) Rhodamine 123, TMRM (Tetramethylrhodamine Methyl Ester) [19], and TMRE (Tetramethylrhodamine Ethyl Ester), which are based on mitochondrial membrane potential [20]. Mito-tracker dyes are still widely used today due to their ability to label live cells and their compatibility with other fluorescent markers [21]. (2) Genetic labeling, such as Mito-GFP, which allows for specific and long-term labeling of mitochondria in living cells [22]. (3) Immunolabeling, utilizing anti-mitochondrial proteins for immunofluorescence and immunohistochemistry, enabling detailed study of mitochondrial distribution and morphology [23]. (4) Chemical probes, such as quantum dot conjugates, which offer advantages over conventional dyes in terms of brightness and light stability, and mitochondria-specific chemical probes like JC-1 [24], which can assess potential changes in the mitochondrial membrane. (5) Advanced imaging techniques, including super-resolution microscopy and live cell imaging, as well as functional imaging with sensors like roGFP and ATeam, which have been developed to monitor redox states and ATP levels in mitochondria, respectively [25], providing real-time insights into mitochondrial function.

Each mitochondrial labeling method has its own set of advantages and disadvantages. For example, early dye-based labeling methods frequently exhibit unstable fluorescence and are limited to live cell labeling. In contrast, genetic tagging methods are operationally complex, requiring high skill levels from experimenters and incurring significant costs. Immunolabeling and quantum dot probes offer strong labeling specificity, bright and stable fluorescence, and are suitable for long-term imaging. However, the labeling process is intricate, and time consuming, resulting in elevated costs. Advanced imaging techniques require expensive microscopy equipment, which may be prohibitive for individual researchers conducting independent mitochondrial labeling experiments. Therefore, there is a demand for a mitochondrial labeling technique that is simple to operate, time-efficient, low-cost, provides bright and stable fluorescence, and does not require expensive equipment. In this study, a new mitochondrial labeling compound, mulberrin-Cy3, along with a corresponding labeling technique, was developed. This compound exhibits bright and stable fluorescence, is cost-effective, user-friendly, time-efficient, safe for human use, and does not require specific environmental temperatures—labeling can be performed at room temperature. This compound and labeling technique provide researchers with a new option for intracellular mitochondrial fluorescence labeling and offer a method for rapid labeling of isolated mitochondria, making visual monitoring of mitochondrial transplantation therapy possible.

## 2. Materials and Methods

Mulberrin was purchased from Chengdu Yirui Biotechnology Co (Chengdu, China). and HeLa cells were preserved in our laboratory. DMEM cell culture medium for HeLa cells was produced by Gibco (Shanghai, China). Penicillin and streptomycin were purchased from Gibco. Mito-Tracker Green (Cat No. C1048), Mito-Tracker Red CMXRos (Cat No. C1049B), ER-Tracker Green (Cat No. C1042S), Golgi-Tracker Green (Cat No. C1045S), and DAPI (Cat No. C1006) cell nuclear staining solution were purchased from Beyotime Biotechnology Co (Shanghai, China). Lyso-Tracker Green (Cat No. S28588) was purchased from Shanghai Yuanye Biotechnology Co (Shanghai, China).

### 2.1. Procedure

#### 2.1.1. Synthesis of Mulberrin-Cy3

Mulberrin-Cy3 was synthesized at Xi’an Qiyue Biotechnology Co (Xi’an, China). using the following detailed steps:

(1). CY3-COOH with a weight of 10 mg was dissolved in 3 mL of DMF (N, N-dimethylformamide), and mulberrin (10.0 eq.), DIC (1,3-diisopropylcarbodiimide) (100.0 eq.), HOBt (1-hydroxybenzotriazole) (1.0 eq.), and DMAP (4-dimethylaminopyridine) (1.0 eq.) were added. This was dissolved completely.
(2). The reaction was stirred under nitrogen protection for 12 h at room temperature and the solvent was removed by spinning under reduced pressure using the eluent dichloromethane/methanol (20:1) and purified by silica gel column chromatography.
(3). Vacuum drying was performed to obtain the mulberrin-Cy3 product.
(4). 3200 QTRAP^®^ LC-MS/MS System was used for mass spectrometric analysis of mulberrin-Cy3.
(5). RF-6000 Spectrofluorometer was utilized for the characterization of mulberrin-Cy3 fluorescence spectra (excitation wavelength, emission wavelength determination, and fluorescence intensity-concentration relationship analysis).

#### 2.1.2. Preparation and Storage of the Mitochondrial Labeling Solution, Mulberrin-Cy3

(1). Weigh 5 mg of mulberrin-Cy3.
(2). Add 2.89 mL of anhydrous ethanol to dissolve mulberrin-Cy3.
(3). Wait for complete dissolution and filter the solution with a 0.22 μM filter tip to remove bacteria, and finally obtain a final concentration of 2 mM mitochondrial labeling solution.
(4). Store the filtered mitochondrial labeling solution in the refrigerator at −20 °C.

### 2.2. Animal (Cancer) Cell Mitochondrial Labeling

(1). HeLa cell culture: HeLa cells should be cultured in Duchenne’s Modified Medium (DMEM) (Gibco, Cat. No. C111885500BT), which consists of 1 g/L D-glucose, L-glutamine, and 110 mg/L sodium pyruvate; add 10% fetal bovine serum (Sangon Biotech, Cat. No. E600001) and 1% penicillin/streptomycin (Gibco, Cat. No. 15140122) to the medium at 37 °C in an incubator (humidity 95% and 5% CO_2_). HeLa cells should be passaged to 12-well cell culture plates and confocal dishes for culture, and the subsequent staining steps can be started when the cell growth density reaches 80%.
(2). Dilute the mitochondrial fluorescent marker master mix to a concentration of 10 μM and add it to the cultured adherent cells. Allow the mixture to incubate at room temperature for 5 min, followed by 3 washes with PBS, each lasting 2 min.
(3). After washing, an appropriate volume of PBS should be added to the cell culture plate wells, and fluorescence microscopy imaging can then be conducted.
(4). HeLa cells should be co-incubated with mulberrin-Cy3 (red fluorescence) and Mito-Tracker Green, with the nuclei subsequently stained using DAPI. After washing with PBS, the cells should be imaged using confocal microscopy.
(5). The mulberrin-Cy3 mitochondrial fluorescent marker in the manner described above can also be used to label isolated mitochondria. In addition, during the staining process described above, mitochondria are usually labeled within 5 min of staining, or fluorescence microscopy and/or laser confocal microscopy can be performed immediately after the addition of the staining solution.

### 2.3. Cellular Mitochondrial Extraction

Refer to the Solarbio Mitochondrial Extraction Kit (Cat No. SM0020) procedure.

(1). Transfer the treated HeLa cells to an ultra-clean bench, remove the medium from the cell culture flasks, wash the flasks twice with 2 mL of PBS solution, then add 1 mL of trypsin to the flasks to digest the cells, shake well so that the trypsin solution fully touches the cells, and then put them into a 37 °C cell culture incubator to digest for 90 s, and then add 2 mL of fresh medium to terminate the digestion, and then gently blow the cells with a 1 mL tip so that the cells are completely detached from the cell wall, and then transfer to a 5 mL centrifuge tube and put it into a centrifuge at 2000 rpm for 5 min. Remove the supernatant at the end of the centrifugation, add 1 mL PBS solution to wash the cells, then transfer to a 1.5 mL centrifuge tube and centrifuge at 2000 rpm for 5 min.
(2). Collect the cells after centrifugation and then process the cell homogenization sample.
(3). Cultured cell homogenization: 5 × 10^7^ cells are needed for each extraction. Add 1 mL of ice pre-cooled Lysis Buffer to resuspend the cells, transfer the cell suspension into a small volume glass homogenizer, and grind 30~40 times in an ice bath.
(4). Transfer the tissue or cell homogenate to a centrifuge tube and centrifuge at 1000× *g* for 5 min at 4 °C.
(5). Remove the supernatant and transfer to a new centrifuge tube, then centrifuge again at 1000× *g* for 5 min at 4 °C.
(6). Remove the supernatant and transfer to a new centrifuge tube and centrifuge at 12,000× *g* for 10 min at 4 °C. The supernatant contains cytoplasmic components from which cytoplasmic proteins can be extracted. Transfer the supernatant to a new centrifuge tube and deposit the mitochondria at the bottom of the tube.
(7). Add 0.5 mL of Wash Buffer to the mitochondrial precipitate to resuspend the mitochondrial precipitate and centrifuge at 1000× *g* for 5 min at 4 °C.
(8). Remove the supernatant, transfer to a new centrifuge tube, centrifuge at 12,000× *g* for 10 min at 4 °C. Discard the supernatant. The high-purity mitochondrial precipitate is at the bottom of the tube.
(9). Resuspend the mitochondrial precipitate with 50–100 μL of Store Buffer or suitable reaction buffer and use immediately for subsequent experiments or store at −70 °C.

### 2.4. Mitochondrial Identification

(1). A total of 2 μL of mitochondrial solution extracted from each treatment group was aspirated onto a slide, covered with a coverslip, and the mitochondrial morphology and fluorescence were observed and photographed using an inverted fluorescence microscope.
(2). An amount of 10 μL of mitochondrial solution extracted from each treatment group was aspirated and added to 100 μL of PBS solution, mixed well, and then transferred to flow tubes for detection on a flow cytometer (URIT Flow Cytometer, BF-700, URIT Medical Electronic Co., Ltd., Guilin, China).
(3). After isolating the mitochondria from mulberrin-treated cells, a portion of the mitochondrial solution was taken and added to mulberrin-Cy3 staining solution (10 μM) and incubated for 5~10 min, and then detected by fluorescence microscopy and flow cytometry (URIT Flow Cytometer, BF-700, URIT Medical Electronic Co., Ltd., Guilin, China).
(4). We extracted mitochondria from mulberrin-Cy3-treated HeLa cells using a mitochondrial extraction kit (Solarbio, Cat No. SM0020, Beijing, China), then extracted mitochondrial proteins. To confirm the successful extraction of mitochondria, we incubated the mitochondrial proteins with a mitochondria-specific tagged protein antibody (COX4I1 antibody, BOSTER, Cat No. A05442-1, Wuhan, China, 1:1000 dilution) and performed Western blotting analysis (WB).

### 2.5. Comparison of Mulberrin-Cy3 with Commercially Available Mitochondrial Labeling Probes

(1). The well-grown HeLa cells were passaged and cultured with confocal cell culture dishes.
(2). When the cell growth density was about 80%, the cells were incubated with mulberrin-Cy3 and Mito-Tracker Green probes; meanwhile, the nuclei of the cells were stained by DAPI.
(3). At the end of the incubation, mitochondrial labeling was photographed on a confocal microscope (Olympus Corporation, Tokyo, Japan). The results were then analyzed.

### 2.6. Determination of the Lowest Concentration of Mulberrin-Cy3 Labeling

There were 8 concentration gradients set up to find the lowest labeling concentration of mulberrin-Cy3 in the mitochondria of HeLa cells. These were 5 nM, 10 nM, 50 nM, 100 nM, 200 nM, 500 nM, 1 μM, and 2 μM. We added the configured mulberrin-Cy3 working solution to the cell wells and incubated them at room temperature for 5 min. After that, we removed the mulberrin-Cy3 staining solution and washed the cells with PBS solution 3 times for 2 min each time. Finally, we added an appropriate amount of PBS solution to cover the cells, preparing them for photography and analysis under the microscope.

## 3. Results

### 3.1. Synthesis Route of the Mitochondrial Red Fluorescent Labeling Compound Mulberrin-Cy3

**Figure 1:**
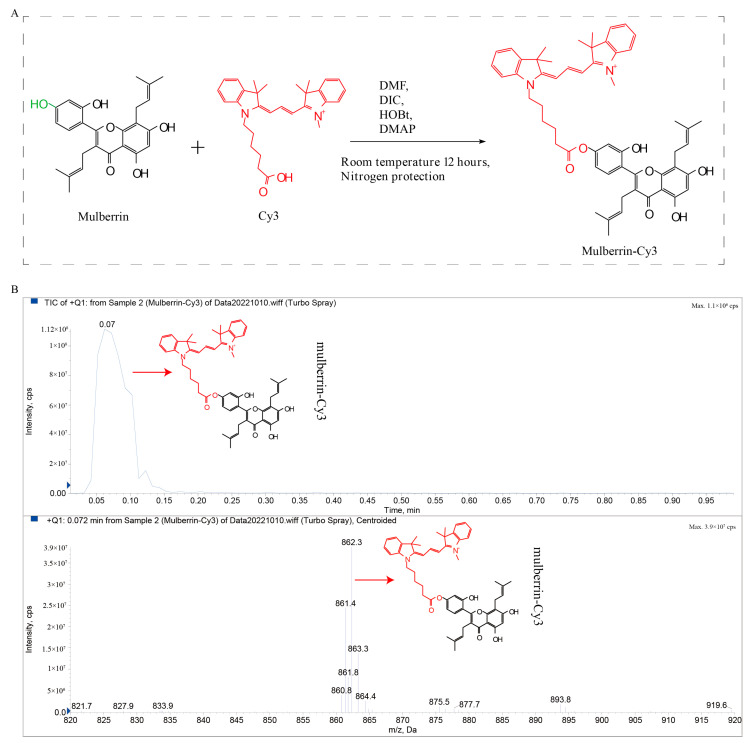
Scheme for the synthesis of mulberrin-Cy3. (**A**) Red fluorescent group CY3 was added to the hydroxyl group of mulberrin’s compound to obtain the new compound mulberrin-Cy3. (**B**) Mulberrin-Cy3 peak detection and mass spectrometry analysis. Abbreviations: DMF (N, N-dimethylformamide), DIC (1,3-diisopropylcarbodiimide), HOBt (1-hydroxybenzotriazole), and DMAP (4-dimethylaminopyridine).

Mulberrin is a natural compound unique to the mulberry tree, and the combination of the red fluorescent group CY3 with the hydroxyl group at the 4′ position of the mulberrin benzene ring yielded the new compound mulberrin-Cy3 (molecular weight 862.09); the synthetic route of mulberrin-Cy3 is shown in Figure 1.

### 3.2. Excitation Wavelength and Emission Wavelength of Mulberrin-Cy3 

**Figure 2:**
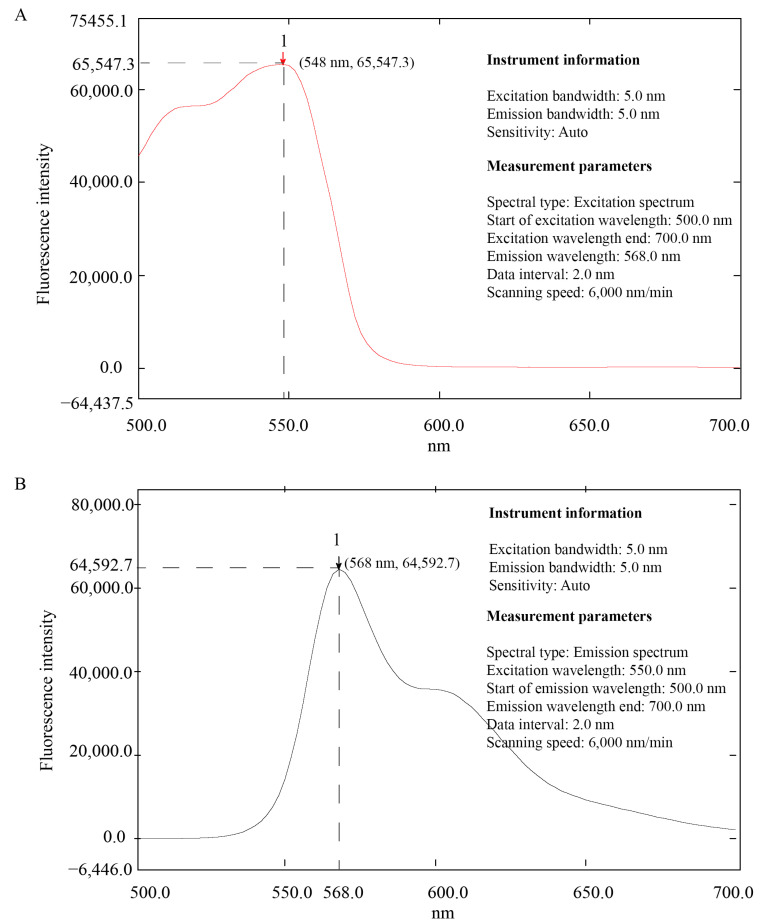
Information on the wavelength of excitation light of mulberrin-Cy3. (**A**) Excitation wavelength and fluorescence intensity of mulberrin-Cy3. (**B**) Emission wavelength and fluorescence intensity of mulberrin-Cy3.

**Figure 3:**
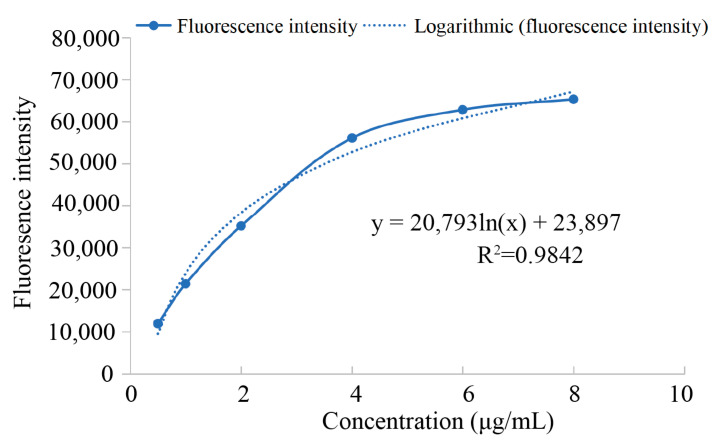
Logarithmic relationship between mulberrin-Cy3 concentration and fluorescence intensity.

The detection results showed that the excitation wavelength of mulberrin-Cy3 was 548 nm (Figure 2A) and the emission wavelength was 568 nm (Figure 2B). According to the statistical results, the concentration of mulberrin-Cy3 and the fluorescence intensity were logarithmically related (Figure 3 and Table 1).

### 3.3. Mitochondrial Labeling of HeLa Cells

**Figure 4:**
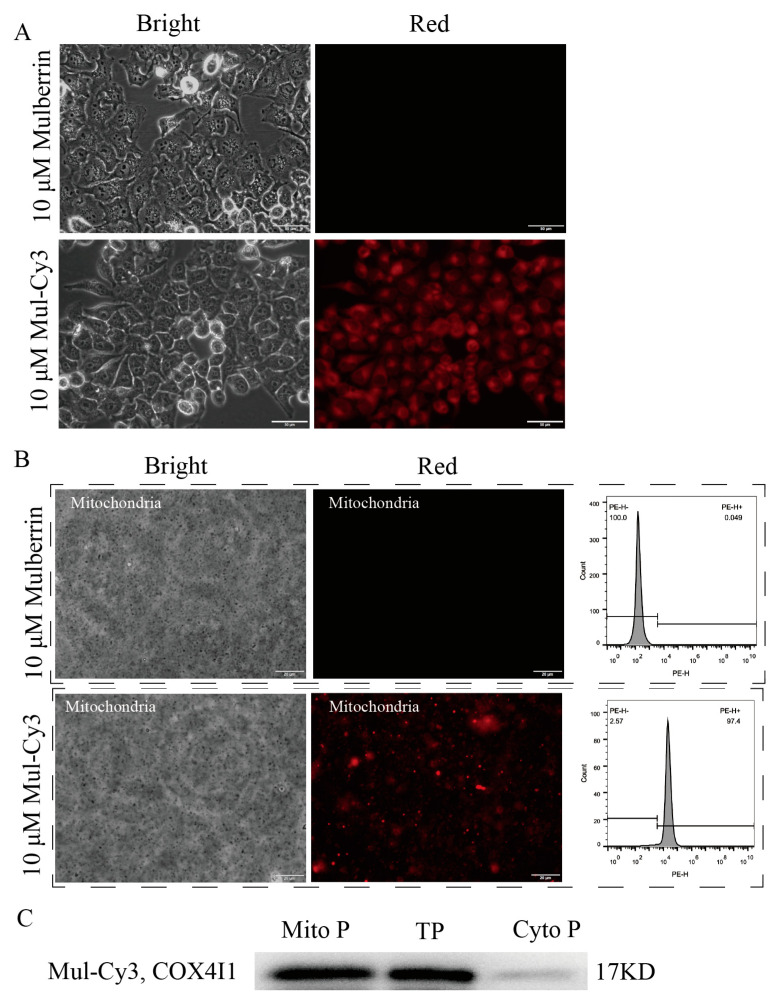
Mulberrin-Cy3 labeling of cellular mitochondria. The top panel (**A**) shows fluorescence micrographs of mulberrin and mulberrin-Cy3 labeled with mitochondria after culture with HeLa cells. Scale bar: 50 μM. The middle panel (**B**) shows the fluorescence labeling of extracted mitochondria after incubation of mulberrin and mulberrin-Cy3 with HeLa cells detected by fluorescence microscopy and flow cytometry. Scale bar: 20 μM. The bottom panel (**C**) shows the identification of extracted mitochondria using the WB method and hybridization with the mitochondria-specific antibody COX4I1. Note: Mito P indicates total mitochondrial protein from extracted HeLa cells, TP indicates total HeLa cell protein, and Cyto P indicates HeLa cell cytoplasmic protein.

When HeLa cells were exposed to mulberrin and mulberrin-Cy3, only the mulberrin-Cy3 group exhibited red fluorescence, while the mulberrin group did not show any red fluorescence (Figure 4A). Mitochondria were isolated from both mulberrin and mulberrin-Cy3-treated HeLa cells. The fluorescent labeling of the mitochondria was then analyzed using fluorescence microscopy and flow cytometry. The results revealed that the extracted mitochondria from the mulberrin-Cy3 group still exhibited red fluorescence, as observed under fluorescence microscopy. Additionally, the flow cytometry analysis demonstrated an increase in the intensity of mitochondrial fluorescence in this group (top of Figure 4B). In contrast, the mitochondria from the mulberrin group did not show any red fluorescence, and the flow cytometry analysis did not detect an increase in mitochondrial fluorescence intensity (bottom of Figure 4B). The Western blotting results for proteins isolated from mitochondria showed stronger COX4I1 bands, indicating the effectiveness of the mitochondrial isolation procedure (Figure 4C).

**Figure 5:**
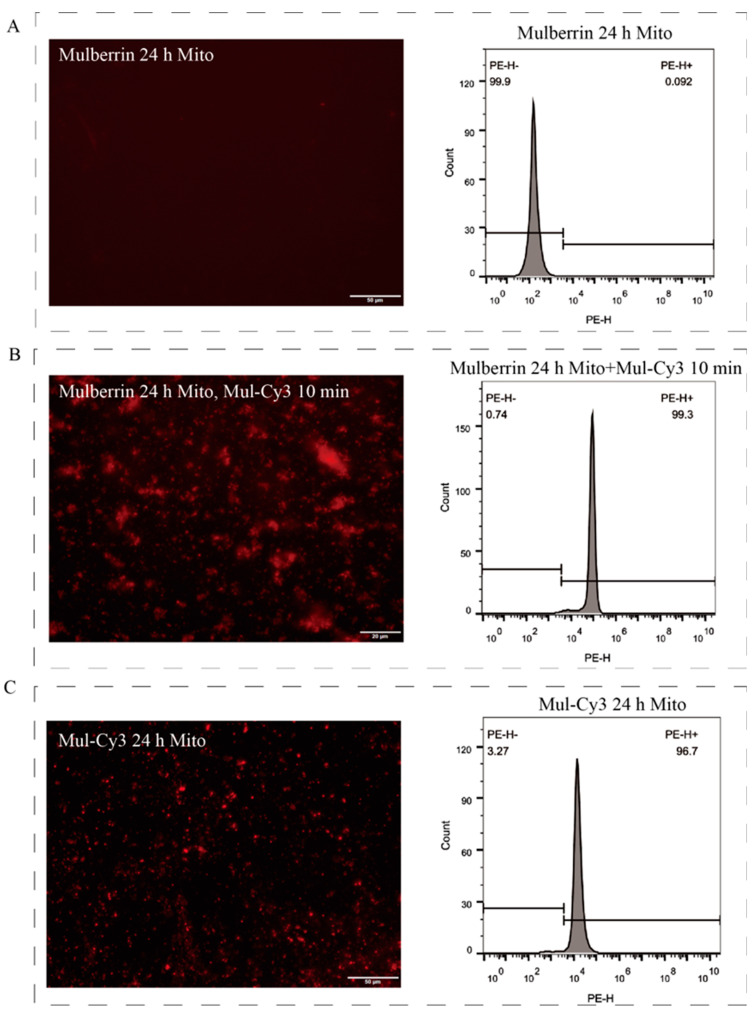
Mitochondrial labeling. (**A**) The figures show the results of mitochondrial extraction after mulberrin treatment of HeLa cells for 24 h: microscopic photograph (left) and flow cytometry assay (right). Scale bar: 50 μM. (**B**) The figures show the mitochondria extracted from HeLa cells after treatment with mulberrin for 24 h and then co-incubation of mulberrin-Cy3 with mitochondria for 10 min: fluorescence microscopic photographing (left) and flow cytometry detection (right) of mitochondrial labeling. Scale bar: 20 μM. (**C**) The figures show the results of the flow cytometry assay of mitochondrial extraction after mulberrin-Cy3 treatment for 24 h: microscopic photograph (left) and flow cytometry assay (right). Scale bar: 50 μM.

To further verify that mulberrin-Cy3 binds directly to mitochondria, mitochondria were isolated from unstained treated HeLa cells using the Mitochondrial Extraction Kit (Figure 5A, Supplementary Movie S1) and then incubated with mulberrin-Cy3 for 10 min. The results of the fluorescence microscopy observation and flow cytometry assay showed that the mitochondria were observed to emit red fluorescence under the microscope, while the results of flow cytometry showed that the fluorescence signal of labeled mitochondria was enhanced (Figure 5B). Meanwhile, after incubating HeLa cells with mulberrin-Cy3 for 24 h, the isolated mitochondria still produced red fluorescence under the microscope, and the flow cytometry assay showed enhanced fluorescence signals of the mitochondria (Figure 5C, Supplementary Movie S2).

### 3.4. Comparison of Mulberrin-Cy3- and Mito-Tracker-Labeled Mitochondria 

**Figure 6:**
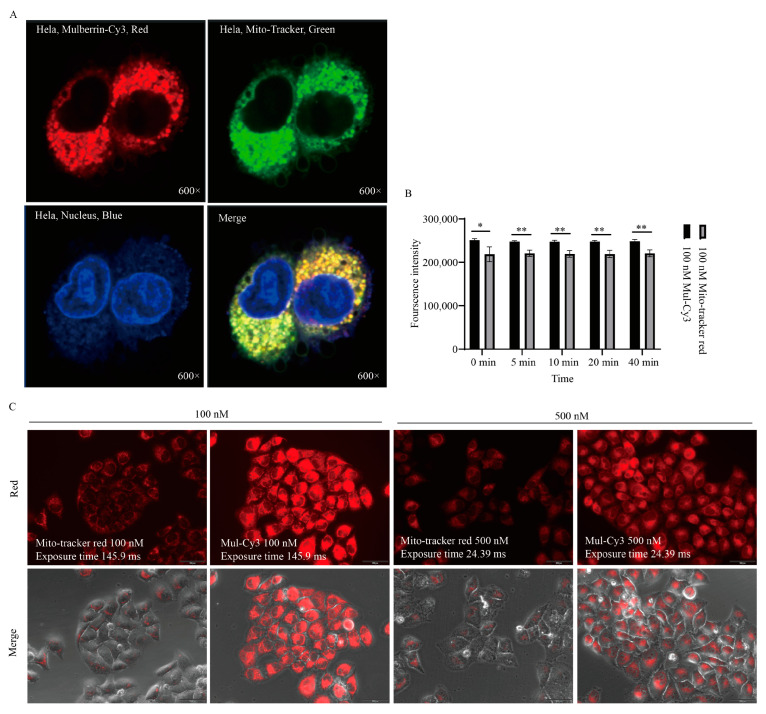
Comparison of mulberrin-Cy3 and Mito-tracker mitochondrial labeling. (**A**) Confocal photographs were taken to compare the labeling of mulberrin-Cy3 and Mito-tracker mitochondria. The mulberrin-Cy3-labeled mitochondria showed red fluorescence, Mito-tracker showed green fluorescence, and the nuclei of the cells were stained with DAPI and showed blue fluorescence. (**B**) Comparison of fluorescence intensity after labeling of HeLa cells with the same concentration of mulberrin-Cy3 and Mito-tracker red. Each bar represents the mean ± SD of three independent experiments. Statistical significance is indicated by * *p* < 0.05, ** *p* < 0.01, (**C**) After labeling cellular mitochondria with 100 nM and 500 nM mulberrin-Cy3 and Mito-tracker red, fluorescence microscopy pictures were taken to compare the intensity of the red fluorescence. Scale bar: 100 μM.

Mulberrin-Cy3 (red fluorescence) and the existing Mito-tracker (green and red fluorescence) were used to label the mitochondria of HeLa cells at the same time to see the difference in labeling. The results showed that mulberrin-Cy3 could label cellular mitochondria as well as Mito-Tracker green and Mito-Tracker red (Figure 6A). In addition, when mulberrin-Cy3 and Mito-tracker red were used to label mitochondria at the same labeling concentration of 100 nM and 500 nM, respectively, the results showed that mulberrin-Cy3-labeled mitochondria could emit brighter red fluorescence, while the red fluorescence intensity of Mito-tracker red was weaker (Figure 6B,C).

### 3.5. Mulberrin-Cy3 Labeling Concentration Measurement

**Figure 7:**
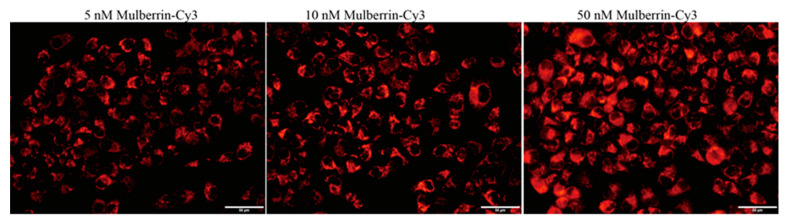
Concentration of mulberrin-Cy3 mitochondrial labeling. Differences in fluorescence intensity between different concentrations of mulberrin-Cy3 labeled mitochondria of HeLa cells. Scale bar: 50 μM.

In order to find the appropriate concentration of mulberrin-Cy3 for mitochondrial labeling, eight concentration gradients were set, from a minimum concentration of 5 nM to a maximum concentration of 2 μM for cellular mitochondrial staining, and the results showed that at the minimum concentration of 5 nM of mulberrin-Cy3, the HeLa cells also emitted red fluorescence under the fluorescence microscope, and with an increase in concentration, the red color of the cells' emitted fluorescence intensity increased (Figure 7). In HeLa cells, considering the better mitochondrial labeling effect, the concentration of mulberrin-Cy3 should not be lower than 5 nM. Depending on different cell lines, mulberrin-Cy3 labeling concentration will vary.

## 4. Discussion

The most commonly used mitochondrial labeling dyes in current experiments are Mito TrackerTM products (rosamine-based Mito Tracker dyes and carbocyanine-based Mito Tracker dyes) [26], although rosamine-based Mito Tracker dyes used to labeled mitochondria were not affected by cell fixatives and the fluorescence labeling of mitochondria was stable after fixative treatment, their cost price was high; the other carbocyanine-based Mito Tracker dyes labeled mitochondria with multicolor labeling, which was easily affected by fixatives and resulted in unstable fluorescence of mitochondria labeling [27]. These problems of cost and instability of mitochondrial labeling dyes are well solved in our newly discovered mitochondrial labeling compound mulberrin-Cy3, which has a low labeling cost and is not affected by fixatives, which is conducive to multicolor labeling.

Mitochondria play a variety of roles in the cell and are essential for numerous biological functions. Mitochondrial abnormalities can lead to disease. Common ailments associated with mitochondrial dysfunction include diabetes [28], neurodegenerative diseases [29], cardiovascular diseases [30], tumors [31], fatty liver [32], and schizophrenia [33]. Advances in research have illuminated the potential of healthy mitochondrial transplantation as a therapeutic strategy for diseases characterized by abnormal mitochondria. Currently, mitochondrial transplantation therapy has been applied to treat central nervous system diseases (such as Parkinson’s, depression, and schizophrenia) [34,35], peripheral system diseases (ischemic myocardial injury, fatty liver, and emphysema) [36], and tumors [37]. However, the rapid monitoring of normal mitochondria entry into cells with mitochondria abnormalities and the assessment of mitochondrial status post-entry have become critical challenges. The newly developed mulberrin-Cy3 mitochondrial labeling compound can rapidly label isolated normal mitochondria, facilitating visual monitoring of mitochondrial transplantation therapy and potentially expediting the therapeutic applications for mitochondrial diseases.

### Advantages of Mulberrin-Cy3

(1) Simple labeling process: do not pre-treat the cells, directly add mulberrin-Cy3 solution to cell culture plates/cell culture flasks (adherent cells) or cell suspension (suspended cells);
(2) Fast labeling, stable red fluorescence, not easy to quench;
(3) Short incubation time (5 min is enough), can be incubated at room temperature;
(4) Stable after staining: it can be labeled with other stains without fear of fixation or permeabilization; it is not necessary to add an anti-fluorescence quencher to prevent fluorescence quenching before taking pictures of the microscope;
(5) Lower cost: the price of developing into a commercial product is much lower than the mainstream products in the current market, which is helpful for reducing the cost of research and development;
(6) The established mulberrin-Cy3 mitochondria staining solution is very stable and can be stored at 4 °C or −20 °C. Repeated freezing and thawing will not affect the mitochondrial labeling effect and it can be stored for a long period of time. Thus, it helps to simplify the sample preparation for cell analysis workflow;
(7) Mulberrin-Cy3 is safe for the human body.

## 5. Conclusions

In summary, the mitochondrial fluorescent labeling compound and method involve a new fluorescent marker obtained by combining a compound unique to mulberry plants, mulberry flavonoid, with a fluorescent group; the fluorescent marker is capable of rapid and specific fluorescent labeling of mitochondria in isolated cells at room temperature. With this labeling method, there is no need to pre-treat the cells during the labeling process, the fluorescence of the marker is stable and not easy to be quenched, and the labeling effect will not be affected by other staining markers after the labeling is completed. This method is characterized by low cost and high safety.

## 6. Patents

The results of this study have been published in a patent in China, Publication No. CN118373812A.

## Figures and Tables

**Table 1:** Mulberrin-Cy3 concentration and fluorescence intensity.

Concentration (μg/mL)	0.5	1	2	4	6	8
Fluorescence intensity	11,944.766	21,375.713	35,119.884	56,129.718	62,816.1	65,311.047

## Data Availability

The original contributions presented in the study are included in the article.

## Supplementary Materials

The following supporting information can be downloaded at: https://www.mdpi.com/article/10.3390/bios14090428/s1, Movie S1: mitochondria movie 1, Movie S2: mitochondria movie 2, S1: Excitation spectrum measurement of mulberrin-Cy3, S2: Emission spectrum measurement of mulberrin-Cy3, S3: Mass spectrum of mulberrin-Cy3, Figure S1: Reactivity analysis of the mulberrin moiety, Figure S2: Fluorescent labeling of cell organelles, Figure S3. Endoplasmic reticulum fluorescence labeling and identification.
