# Supplementary material for: Mitochondrial Labeling with Mulberrin-Cy3: A New Fluorescent Probe for Live Cell Visualization
Source: Biosensors (Basel). 2024 Sep 5;14(9):428. doi: 10.3390/bios14090428 (PMC11429601; doi:10.3390/bios14090428)
Supplement: Supplementary file 1 [file biosensors-14-00428-s001.zip › S1 Excitation spectrum measurement of mulberrin-Cy3.pdf]

Excitation spectrum measurement of mulberrin-Cy3

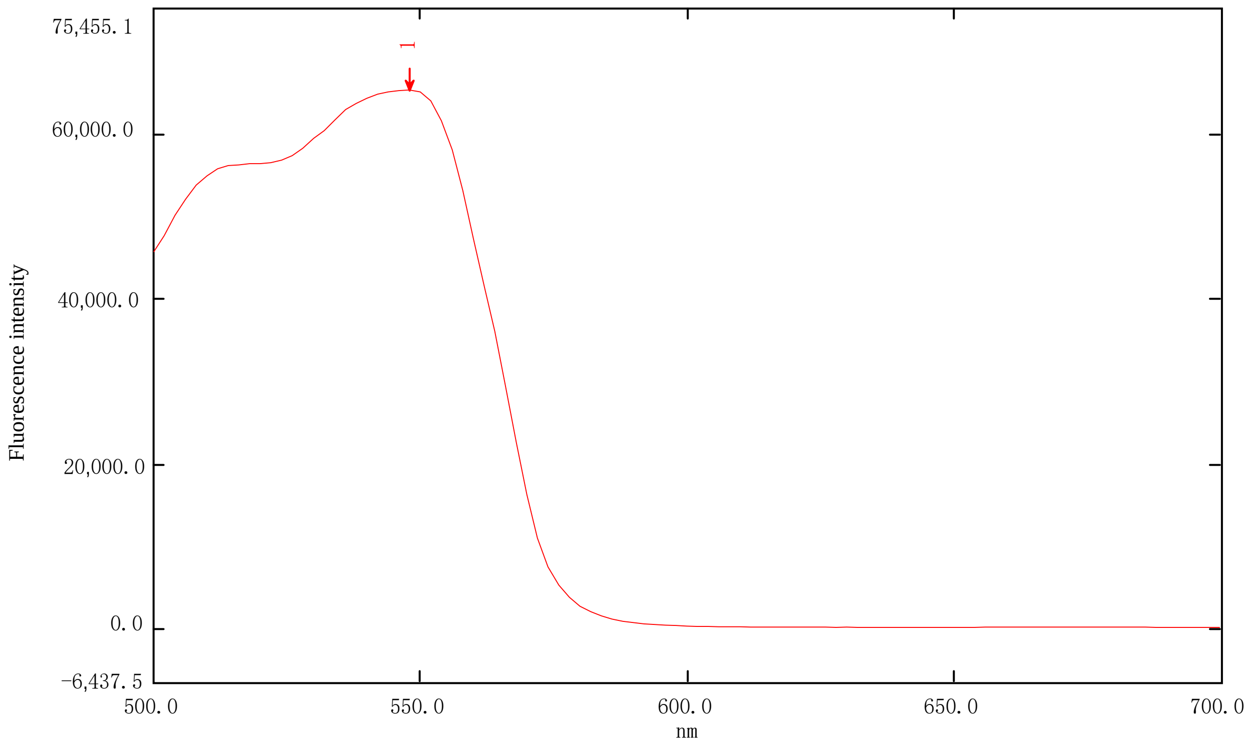

Creation Date: 2022/10/27 18:58:03

Sample Name: Mulberrin-Cy3

Instrument Name: RF-6000

Instrument Model: RF-6000 series

[Assay]

Spectral type: Excitation spectrum

Excitation wavelength, Start: 500.0 nm

Excitation wavelength, End: 700.0 nm

Emission wavelength: 568.0 nm

Data interval: 2.0 nm

Scanning speed: 6,000 nm/min

[Peak Detection Table]

Threshold Value: 1,000, 000

Check Numbers: 5

No.	P/V	Wavelengt	FI
1	↑	548.0	65547.3

[Instrument information]

Excitation bandwidth: 5.0 nm

Emission bandwidth: 5.0 nm

Sensitivity: Auto
