# Supplementary material for: Mitochondrial Labeling with Mulberrin-Cy3: A New Fluorescent Probe for Live Cell Visualization
Source: Biosensors (Basel). 2024 Sep 5;14(9):428. doi: 10.3390/bios14090428 (PMC11429601; doi:10.3390/bios14090428)
Supplement: Supplementary file 1 [file biosensors-14-00428-s001.zip › S2 Emission spectrum measurement of mulberrin-Cy3.pdf]

Emission spectrum measurement of mulberrin-Cy3

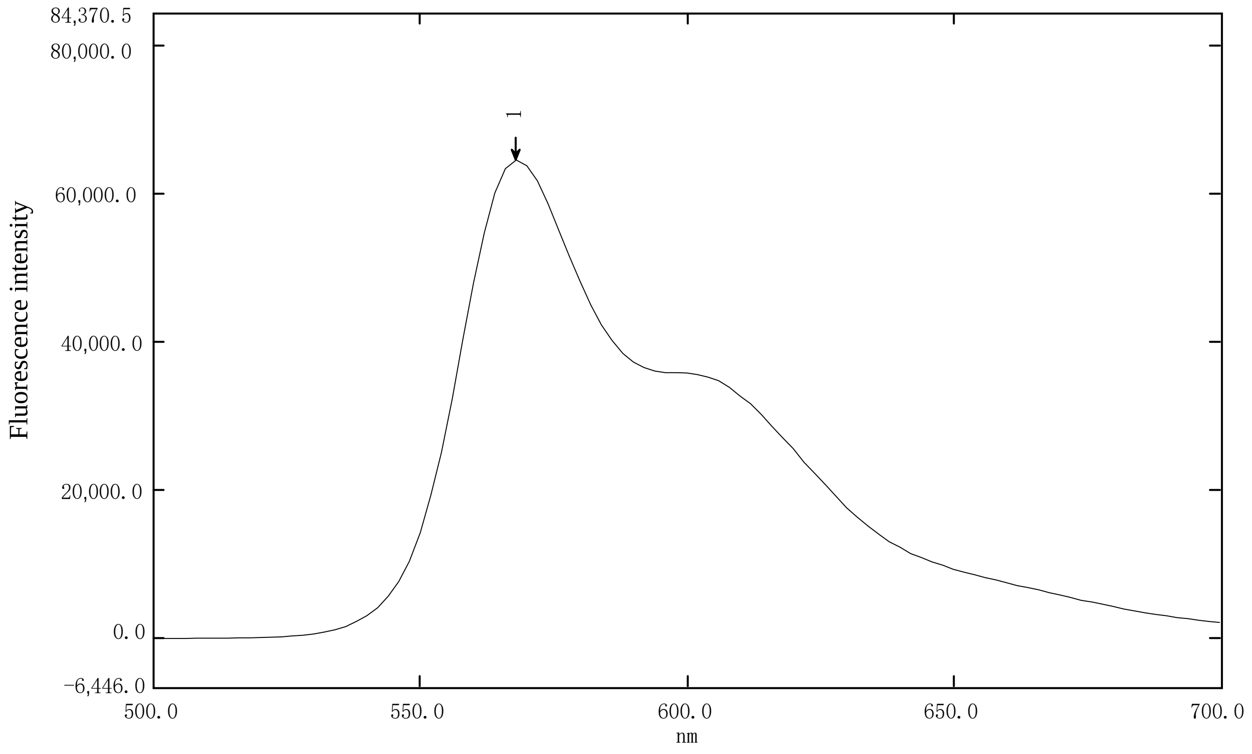

Creation Date: 2022/10/27 18:57:05
Sample Name: Mulberrin-Cy3
Instrument Name: RF-6000
Instrument Model: RF-6000 series

[Peak Detection Table]

Threshold Value: 1,000, 000
Check Numbers: 5

No.	P/V	Wavelengt	FI
1	↑	568.0	64,592.7

[Assay]

Spectral type: Emission spectrum
Excitation wavelength: 550. 0 nm
Emission wavelength, Start: 500. 0 nm
Emission wavelength, End: 700. 0 nm
Data interval: 2. 0 nm
Scanning speed: 6,000 nm/
min

[Instrument information]

Excitation bandwidth: 5.0 nm
Emission bandwidth: 5.0 nm
Sensitivity: Auto
