# Supplementary material for: Mitochondrial Labeling with Mulberrin-Cy3: A New Fluorescent Probe for Live Cell Visualization
Source: Biosensors (Basel). 2024 Sep 5;14(9):428. doi: 10.3390/bios14090428 (PMC11429601; doi:10.3390/bios14090428)
Supplement: Supplementary file 1 [file biosensors-14-00428-s001.zip › S4-Supplementary figure.pdf]

Supplementary Figures

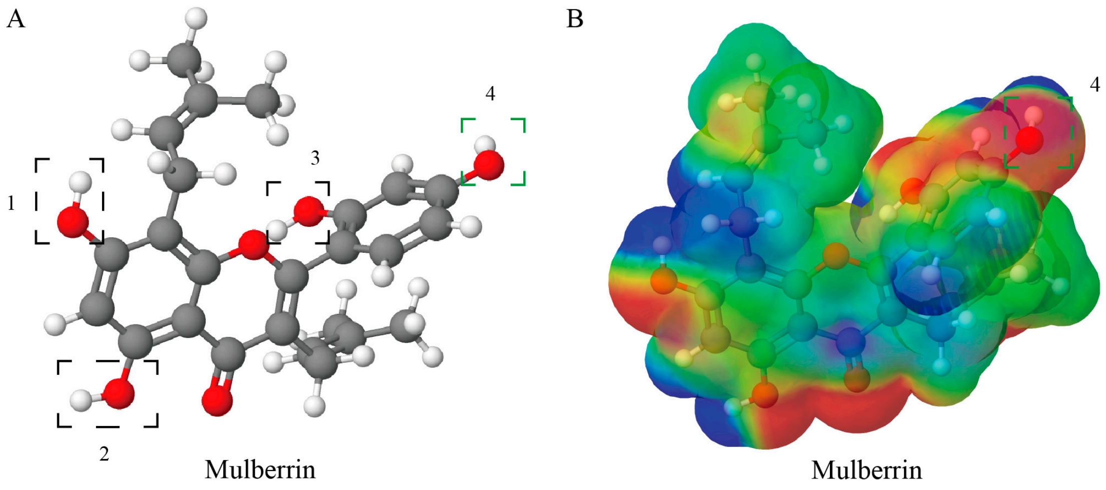

Figure S1. Reactivity analysis of the mulberrin moiety. (A) Mulberrin's 3D structure. The dashed box marks the locations of the four hydroxyl groups of mulberrin. (B) Using MolView to analyze the molecular electrostatic potential of mulberrin (red indicates negative potential regions, related to electrophilic reaction sites; blue indicates positive potential regions, representing suitable centers for nucleophilic attack).

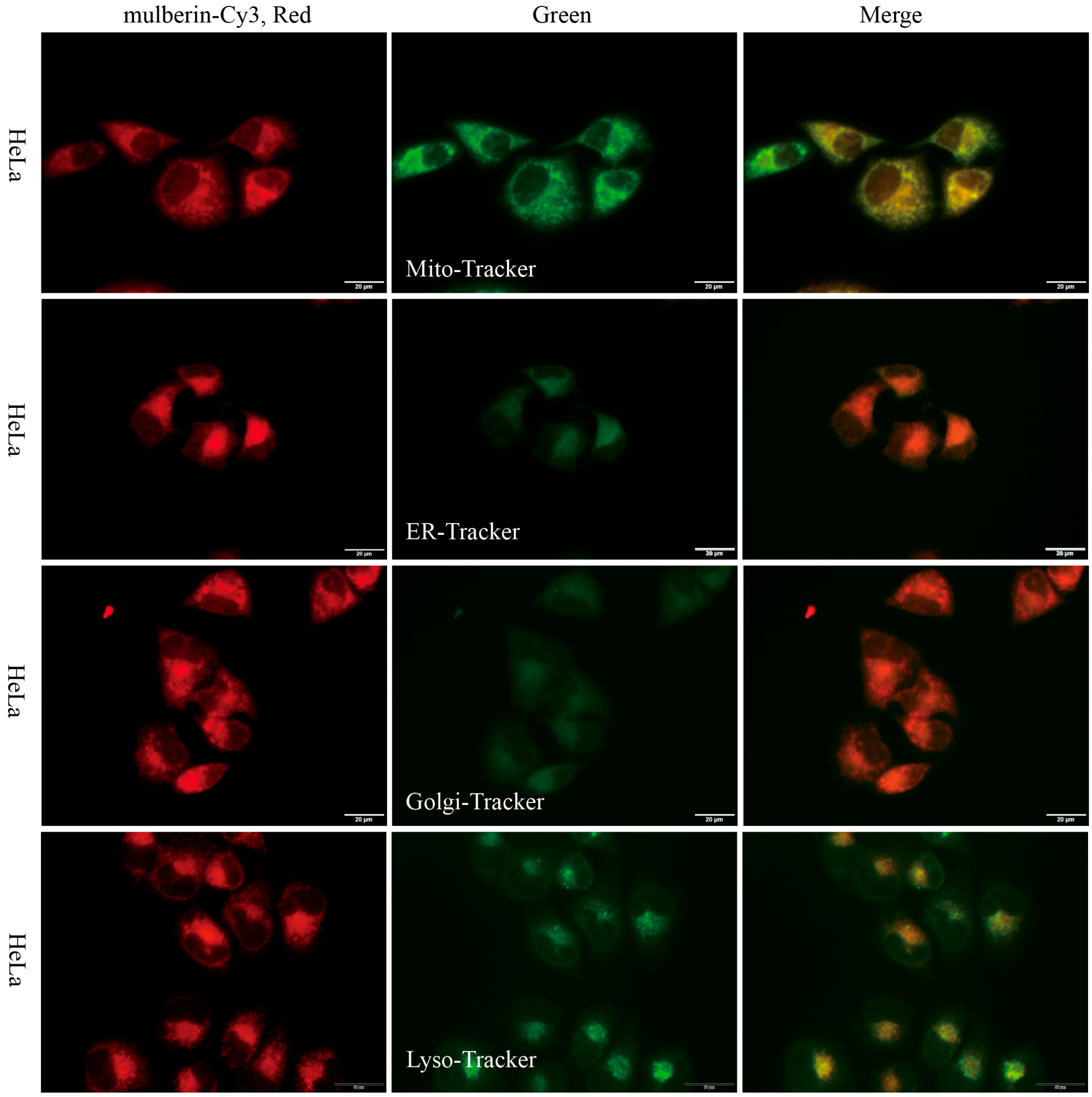

Figure S2. Fluorescent labeling of cell organelles. Co-localization analysis was performed with fluorescently labeled probes for mitochondria, endoplasmic reticulum, Golgi apparatus, and lysosomes, respectively, with mulberrin-Cy3 fluorescent dye.

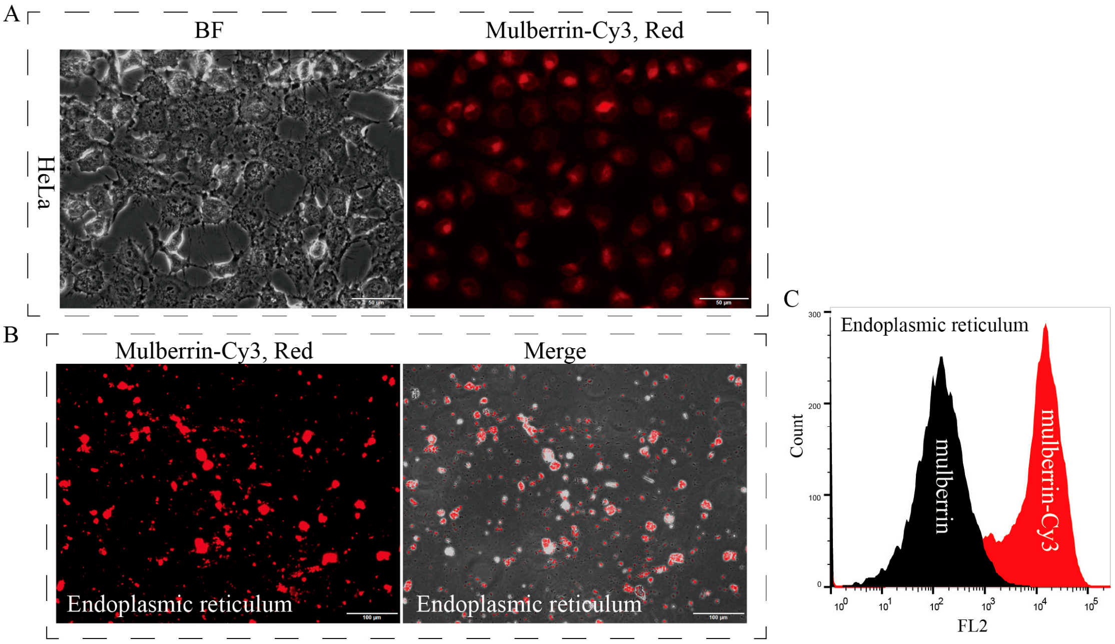

Figure S3. Endoplasmic reticulum fluorescence labeling and identification. (A) The image displays the fluorescent labeling of Mulberrin-Cy3 following its co-incubation with HeLa cells. Scale bar: 50 µM. (B) Fluorescent photograph of HeLa cells after incubation with Mulberrin-Cy3 and after isolation of the endoplasmic reticulum. Scale bar: 100 µM. (C) Extraction of endoplasmic reticulum fluorescence labeling by flow cytometry assay.
