# Supplementary figures and images for: Mitochondrial Labeling with Mulberrin-Cy3: A New Fluorescent Probe for Live Cell Visualization
Source: Biosensors (Basel). 2024 Sep 5;14(9):428. doi: 10.3390/bios14090428 (PMC11429601; doi:10.3390/bios14090428)

Mass spectrum of mulberrin-Cy3

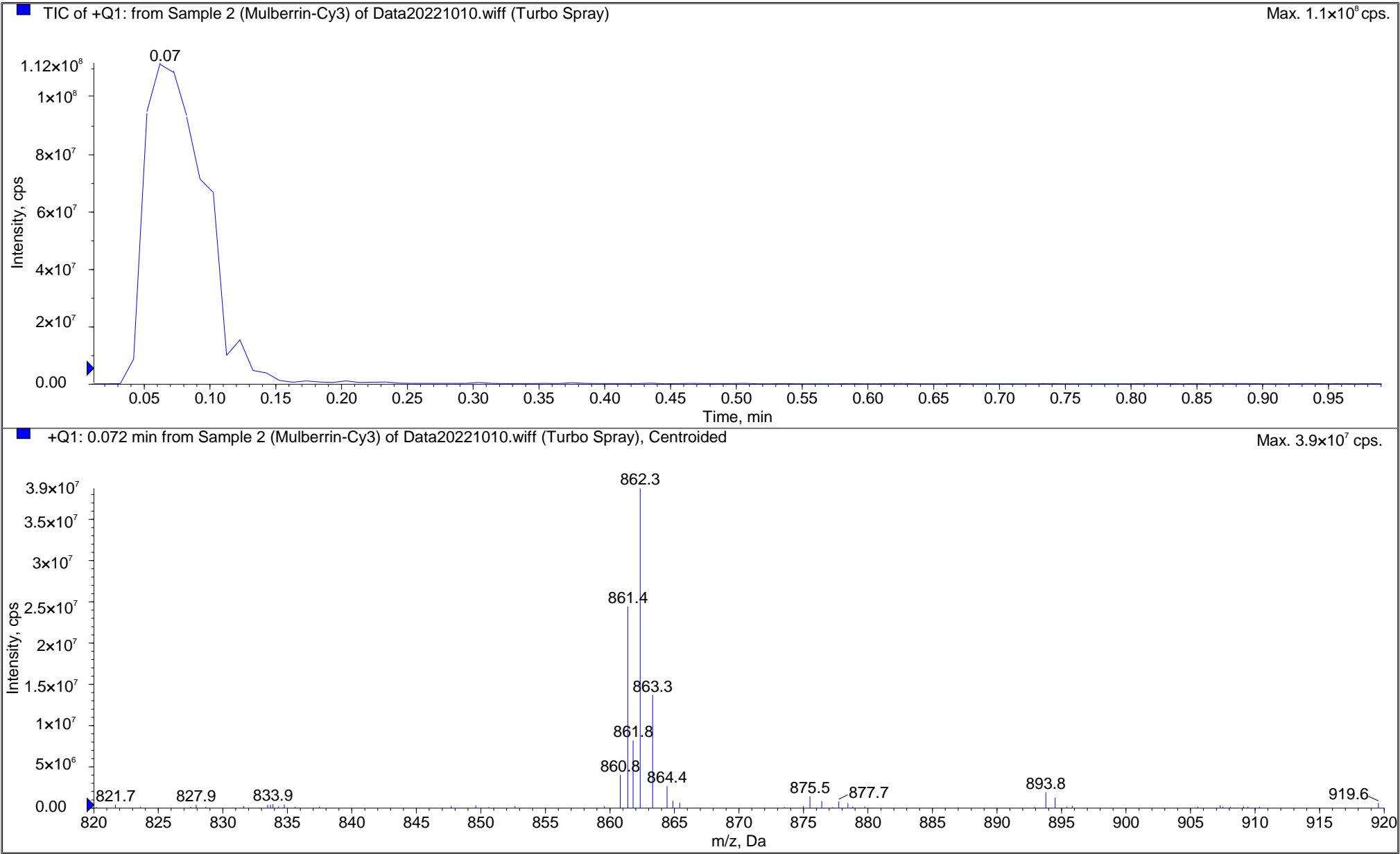

Supplement: Supplementary file 1 [file biosensors-14-00428-s001.zip › S3 Mass spectrum of mulberrin-Cy3.pdf]
